# Cumulative environmental quality is associated with breast cancer incidence differentially by summary stage and urbanicity

**DOI:** 10.1038/s41598-023-45693-0

**Published:** 2023-11-20

**Authors:** Larisa M. Gearhart-Serna, Brittany A. Mills, Hillary Hsu, Oluwadamilola M. Fayanju, Kate Hoffman, Gayathri R. Devi

**Affiliations:** 1 Department of Surgery, Duke University School of Medicine, DUMC Box 2606 Med Ctr, Durham, NC 27710 USA; 2 Department of Pathology, Duke University School of Medicine, Durham, NC USA; 3 Nicholas School of the Environment, Duke University, Durham, NC USA; 4 Duke Consortium for Inflammatory Breast Cancer, Duke Cancer Institute, Durham, NC USA; 5 Trinity College of Arts and Sciences, Duke University, Durham, NC USA; 6 Department of Surgery, Perelman School of Medicine, University of Pennsylvania, Philadelphia, PA USA

**Keywords:** Breast cancer, Environmental sciences

## Abstract

Individual environmental contaminants have been associated with breast cancer; however, evaluations of multiple exposures simultaneously are limited. Herein, we evaluated associations between breast cancer summary stages and the Environmental Quality Index (EQI), which includes a range of environmental factors across five domains. The EQI (2000–2005) was linked to county-level age-standardized incidence rates (SIRs) obtained from the North Carolina Central Cancer Registry (2010–2014). Incidence rates and SIRs of total, in situ, localized, regional, and distant breast cancers were evaluated stratified by rural–urban status. In counties with poor environmental quality compared to those with good environmental quality, total breast cancer incidence was higher by 10.82 cases per 100,000 persons (95% CI 2.04, 19.60, p = 0.02). This association was most pronounced for localized breast cancer (β = 5.59, 95% CI 0.59, 10.58, p = 0.03). Higher incidence of early-stage disease (carcinoma in situ β = 5.25, 95% CI 2.34, 8.16, p = 0.00 and localized breast cancer β = 6.98, 95% CI 2.24, 11.73, p = 0.00) and total breast cancer (β = 11.44, 95% CI 3.01, 19.87, p = 0.01) occurred in counties with poor land quality, especially urban counties. Our analyses indicate significant associations between environmental quality and breast cancer incidence, which differ by breast cancer stage and urbanicity, identifying a critical need to assess cumulative environmental exposures in the context of cancer stage.

## Introduction

Breast cancer is the most common and second most lethal invasive cancer in women in the U.S.^1^ Breast cancer risk is shown to be impacted by a combination of both genetic and environmental factors^2^ including demographics such as age, race, reproductive age or history, weight, income, and location^3–7^, while survival can be impacted by the resulting tumor stage, morphology, histology, gene expression, and hormone receptor status^3,8–11^. Increasing evidence highlights environmental factors such as tobacco smoke^12^, pesticides^13–16^ and insecticides^17^, and bisphenol-A food contamination^18^ which are associated with an increased risk of breast cancer^19–21^. While these studies demonstrate links between specific environmental exposures and breast cancer development, many studies including ours using preclinical cancer models^22,23^ have primarily focused on exposure to a single contaminant or a small class of environmental contaminants. In addition, these study methodologies lack the ability to elucidate the combined effects of the chemical, biological and social factors encountered on a daily basis. Failing to consider real-world exposure scenarios, which often occur in mixtures and simultaneously, may underestimate the true impacts of the environment on breast cancer incidence.

The U.S. Environmental Protection Agency (USEPA) constructed an environmental quality index (EQI), which contains county-level environmental exposure data across five overarching environmental domains^24–26^. The EQI was created to help researchers better understand health and disease outcomes and how they may be associated with cumulative environmental exposures. The EQI has previously been used to study associations with health outcomes such as birth defects, diabetes, end-stage renal disease, asthma, infant mortality, preterm birth, and obesity, as well as overall mortality^27–35^. The utility of the EQI includes not only the quantity of accumulated data, but also the focused stratification of factors into different environmental domains, as well as stratification by county urbanicity. This allows researchers to take a high-level look at broader environment and health outcome associations, such as prior research linking the EQI to several cancer types, including breast cancer. As part of this broad cancer and environment assessment across the US, Jagai et al. revealed a significant association between total breast cancer incidence and poor environmental quality as measured by the EQI^36^. In particular, breast cancer was positively associated with poor air, built environment, and sociodemographic environment quality, and these associations varied by rural–urban strata.

Unfortunately, the links made between breast cancer and the EQI are lacking because these analyses used total breast cancer incidence quantities, masking potential associations between EQI domains and specific stages of disease. This is a problem, as breast cancer is a highly heterogeneous disease with many known risk factors that differ by disease stage and hormone receptor subtype identified at diagnosis, which in turn impacts clinical outcomes^37,38^. In a 2020 study^39^, we set out to address this issue by investigating the impact of the EQI, across all domains and urbanicities, on the odds of a breast cancer patient having later stage invasive disease compared to non-invasive carcinoma in situ. This elucidated the associations between cumulative environmental exposures and breast tumor invasiveness. In this study, we continued along the path of our previous work and investigated if the EQI was associated with an increased incidence of different breast cancer stages, to determine whether environmental quality and urbanicity are related to carcinogenesis and the development of more advanced tumors in a community.

## Methods

### Environmental Quality Index

The EQI is a publicly available county-level measure of cumulative environmental exposures, reported in quantities as part of the “total” environment as well as for five different environmental domains—air, water, land, sociodemographic, and built environments. The EQI (2000–2005) was constructed by the USEPA in four distinct steps: 1. identification of environmental domains, 2. identification of sources of data from 2000 to 2005 for individual factors that would make up each domain, 3. constructing variables based on these data, and reduction of data including compilation into domain-specific entities and 4. computing a total EQI score. The EQI was developed for all U.S. counties, and further accounts for environmental differences by rural–urban context by grouping counties into rural–urban continuum codes. Specific environmental factors which make up each domain can be found in the 2014 USEPA Environmental Quality Index Overview Report^24^. Some examples of included factors are PM2.5 for the air domain, mercury pollution for water, fungicide application for land, crime levels for sociodemographic, and highway safety for built^25,26^.

### Incidence rates of breast cancer in North Carolina counties

The North Carolina Central Cancer Registry (NC CCR) is a reporting system for all cancer cases diagnosed in residents of the state of North Carolina. Data is made available to both the public and for research purposes with appropriate patient protections for the collection and analysis of cancer patient data, including breast cancer. All study related protocols were reviewed and approved by the Duke University Medical Center Institutional Review Board. For these analyses, NC CCR provided diagnoses of breast cancer in all 100 North Carolina counties. We focused on breast cancer cases diagnosed between 2010 and 2014 to account for a 10-year lag time between EQI exposures and breast cancer diagnoses. Breast cancer diagnoses were analyzed in total and were also classified as carcinoma in situ, localized, regional, or distant breast cancer based on summary staging definitions from the Surveillance, Epidemiology, and End Results program of the National Cancer Institute (SEER) (Supplemental Table S1). For consistency with how cases are reported in the NC CCR, “total breast cancer” is defined as the sum of all SEER summary stages carcinoma in situ, localized, regional, and distant breast cancer. Specific counties in NC were excluded from stage-specific analyses if they reported < 5 cases per year, due to unstable rate estimates and no calculated incidence rate. This was true for 5 counties when analyzing carcinoma in situ (all rural counties) and 22 counties when analyzing distant breast cancer (19 rural and 3 urban counties). County-level annual age-adjusted incidence rates were calculated using patient case numbers and U.S. Census 2010 population estimates by county for age categories 0–19, 20–44, 45–64, and 65 and above. Rates were calculated for each breast cancer summary stage. The overall and domain-specific EQI values (2000–2005) were matched to county-level annual age-adjusted cancer incidence rates for analyses.

### Standardized incidence ratios (SIRs)

Standardized incidence ratios (SIRs) comparing age-standardized county-level incidence rates for total, in situ, localized, regional, and distant breast cancers to NC state-wide incidence rates were calculated using U.S. 2010 Census population estimates for each county and each breast cancer summary stage. SIRs were mapped using ArcGIS 10.5.1, with county-level SIR data providing quantities for categorization and visualization.

### Data and statistical analysis

EQI data specifically for North Carolina counties from the 2000–2005 dataset were dichotomized at the median, representing a “good” environmental quality category (1st and 2nd quartiles) and a “poor” environmental quality category (3rd and 4th quartiles). This was done to ensure sufficient n within the categories and thus ensure statistical capability to detect significance. Associations between county-level age-adjusted cancer incidence rates for each summary stage were assessed using general linear models (SAS 9.3), linear models with a continuous outcome with a p-value cutoff for statistical significance set at p < 0.05. These models compared environmental quality using the “good” environmental quality as the reference and results are reported as estimated incidence rate increase for counties with “poor” environmental quality. Due to the significant impacts of factors such as race, age, and mammography screening rates on breast cancer incidence revealed in prior research^40,41^, we evaluated county percentage of smokers and percent African American, from the U.S. Census 2010 estimates for 2014 population, as well as mammography screening rates, from the National Cancer Institute’s state cancer profiles for women of all races, ages 40 +, who had a mammogram in the past two years (year 2014) in bivariate analyses. Results of these analyses were used to inform the selection of confounding variables for EQI analyses. For simplicity, all EQI analyses included the same covariates (e.g., if county percent African American was associated with any breast cancer summary stage, it was included in models for all summary stages). Domain-specific models were further adjusted for all other EQI domains.

### Rural–urban sensitivity analysis

Urbanicity has previously been associated with spatial variation of disease, including breast cancer^42–45^. We first evaluated incidence rates by summary stage comparing across rural versus urban counties using Mann–Whitney rank tests, since not all stages were normally distributed, confirmed by the D'Agostino-Pearson normality test. To evaluate potential differences in the impact of environmental quality of breast cancer incidence in urban and rural communities, we conducted sensitivity analyses stratifying the previously quartiled EQI values by county urbanicity. These strata were dependent on each county’s rural urban continuum code (RUCC)^46^ as defined in the EQI, which consolidated the original nine RUCCs into four RUCCs. In this study, RUCCs were further consolidated from four to two categories to ensure sufficient n for statistical analyses. The “urban” category was defined as the combination of EQI RUCC1 metropolitan urbanized and RUCC2 non-metro urbanized, which describes anywhere from nonmetro counties with urban population of 20,000 or more, not adjacent to a metro area to counties in metro area with 1 million population or more. Conversely, the “rural” category combined EQI RUCC3 less urbanized and RUCC4 thinly populated, which describes anywhere from nonmetro county completely rural or less than 2,500 urban population, not adjacent to metro area to nonmetro county with urban population of 2500–19,999, adjacent to a metro area.

## Results

### Patterns of county-level incidence ratios vary by breast cancer summary stage

**Figure 1:**
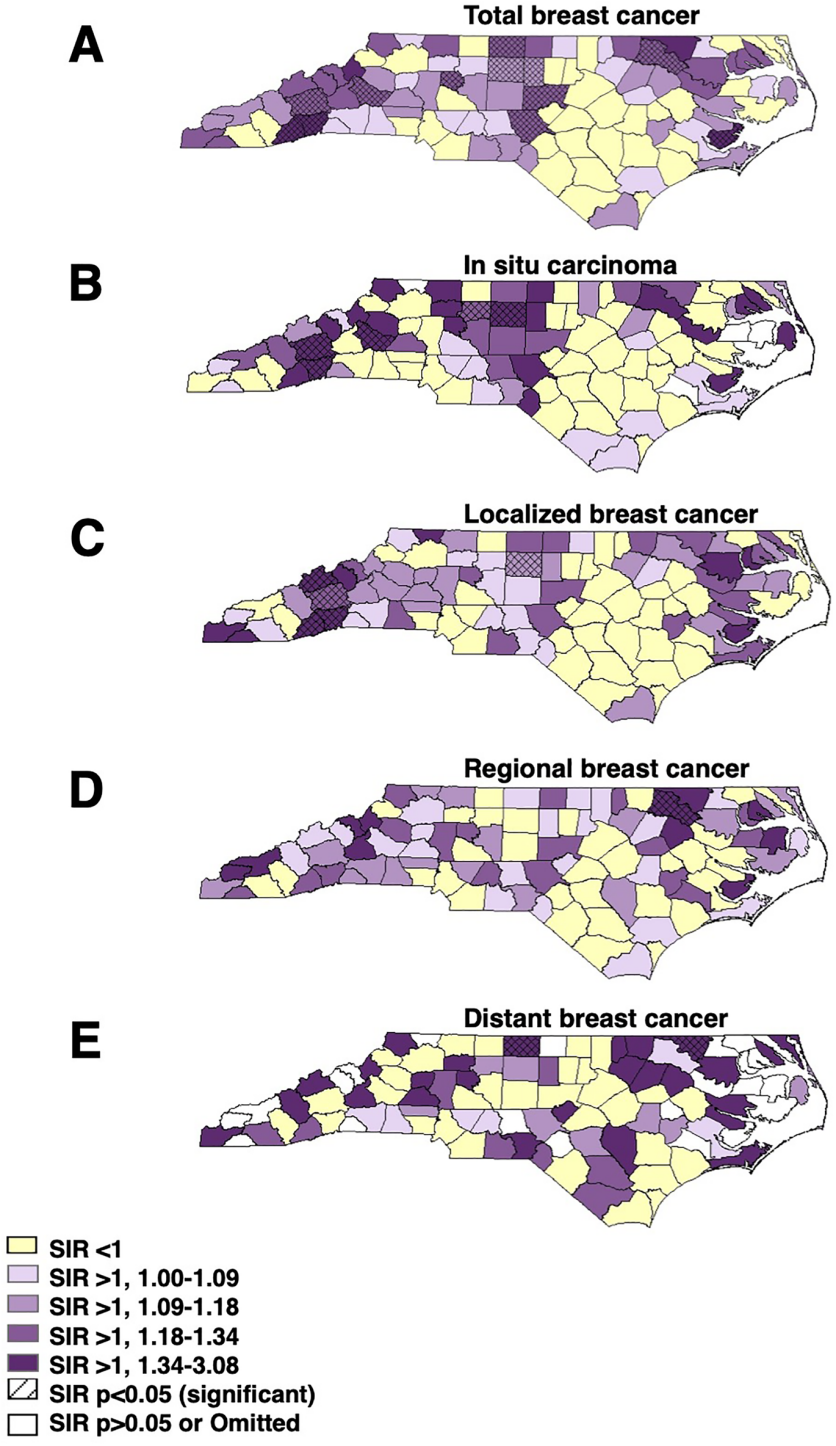
Significantly High Standardized Incidence Ratios by County. Quartiled standardized incidence ratios (SIRs) compared to NC statewide incidence for (**A**) total, (**B**) in situ carcinoma, (**C**) localized, (**D**) regional, and (**E**) distant breast cancer in North Carolina, 2010–2014. Data sourced from the NC Central Cancer Registry, adjusted to the US 2010 Census.

Incidence rates for total breast cancer averaged 153.5 cases per 100,000 persons in North Carolina between years 2010–2014. The vast majority of breast cancer cases were localized (54%), followed by regional (26%), in situ (16%), and distant (4%). All summary stages of breast cancer tended to have higher incidence in the northeastern region of the state (Supplemental Fig. S1), while in situ had a much higher incidence region in central NC. Additionally, incidence of carcinoma in situ and localized breast cancer tended to be higher in the western portion of the state. Counties with the highest distant breast cancer incidence were dispersed throughout the state. Likewise, counties with significantly high SIRs varied across the state and by breast cancer summary stage (Fig. 1).

### Breast cancer incidence varies by county urbanicity

Urbanicity is known to vary across the state of North Carolina and can substantially impact both breast cancer incidence rates and environmental quality. In this analysis, there were 57 NC counties classified as urban, and 43 classified as rural. Interestingly, average incidence per 100,000 persons was higher in urban counties for total (157.2 urban, 148.7 rural), carcinoma in situ (25.5 urban, 25.0 rural), and localized breast cancers (84.3 urban, 80.5 rural), while average incidence was higher in rural counties for regional (40.7 urban, 41.5 rural) and distant breast cancers (7.1 urban, 8.4 rural) (Supplemental Table S2). However, this rural–urban divide was only statistically significant for total breast cancer (p = 0.049, Mann–Whitney test), but approached significance for distant breast cancer (p = 0.074).

### County-level African American population, mammography screening rate, and smoking population associated with breast cancer incidence

Overall, North Carolina counties averaged 21.6% percent African American^45^ which is higher than the US national average of 12.6% at the time of the U.S. 2010 Census^47^. It is interesting to note that the percentage of African Americans in each county in bivariate analyses was associated with increased incidence of regional (incident cases 0.12 cases per % increase in African American population, 95% CI 0.01, 0.22, p = 0.02) and distant breast cancers (incident cases 0.06 per % increase in African American population, 95% CI 0.02, 0.10, p = 0.00) (Table 1). In addition, in stratified models, associations persisted and were strengthened in urban county models for regional breast cancer and in rural county models for distant breast cancer.

**Table 1:** Generalized linear model estimates and associated p-values for county characteristics, per 1% increase.

	Non-stratified		Urban		Rural	
	Estimate (95% CI)	p-value	Estimate (95% CI)	p-value	Estimate (95% CI)	p-value
Total						
County characteristics						
Percent smokers	− 0.74 (− 2.97, 1.48)	0.51	− 2.16 (− 4.76, 0.42)	0.10	0.57 (− 4.08, 5.23)	0.80
Percent African American	0.12 (− 0.16, 0.40)	0.41	0.29 (− 0.06, 0.65)	0.11	− 0.04 (− 0.54, 0.45)	0.85
Mammography screening rate	0.26 (− 0.42, 0.95)	0.45	− 0.64 (− 1.60, 0.30)	0.18	0.80 (− 0.31, 1.91)	0.15
In situ						
County characteristics						
Percent smokers	− 0.23 (− 1.05, 0.59)	0.58	− 0.46 (− 1.59, 0.67)	0.42	0.18 (− 1.30, 1.67)	0.80
Percent African American	− 0.08 (− 0.19, 0.02)	0.11	− 0.12 (− 0.27, 0.03)	0.13	− 0.09 (− 0.28, 0.08)	0.30
Mammography screening rate	0.11 (− 0.20, 0.44)	0.47	− 0.05 (− 0.47, 0.36)	0.79	0.34 (− 0.25, 0.94)	0.25
Localized						
County characteristics						
Percent smokers	− 1.14 (− 2.41, 0.12)	0.08	− 1.33 (− 2.79, 0.11)	0.07	− 0.81 (− 3.60, 1.97)	0.56
Percent African American	0.11 (− 0.05, 0.27)	0.19	0.13 (− 0.07, 0.33)	0.20	0.07 (− 0.22, 0.37)	0.59
Mammography screening rate	0.04 (− 0.35, 0.43)	0.83	− 0.09 (− 0.63, 0.43)	0.72	0.16 (− 0.50, 0.83)	0.62
Regional						
County characteristics						
Percent smokers	− 0.27 (− 1.09, 0.54)	0.50	− 0.63 (− 1.43, 0.15)	0.11	− 0.10 (− 1.94, 1.73)	0.91
Percent African American	**0.12 (0.01, 0.22)**	**0.02**	**0.26 (0.15, 0.37)**	**0.00**	0.03 (− 0.16, 0.22)	0.76
Mammography screening rate	**− 0.31 (**− **0.57, **− **0.06)**	**0.02**	− **0.47 (**− **0.76, **− **0.18)**	**0.00**	− 0.19 (− 0.63, 0.24)	0.38
Distant						
County characteristics						
Percent smokers	0.02 (− 0.28, 0.33)	0.88	0.29 (− 0.01, 0.61)	0.06	− 0.47 (− 1.26, 0.32)	0.23
Percent African American	**0.06 (0.02, 0.10)**	**0.00**	0.01 (− 0.03, 0.05)	0.55	**0.14 (0.05, 0.22)**	**0.00**
Mammography screening rate	0.12 (0.00, 0.24)	0.05	0.06 (− 0.06, 0.18)	0.33	0.20 (− 0.06, 0.48)	0.12

Results are stratified by breast cancer stage and urbanicity. Bolded text indicates statistically significant estimates (p < 0.05).

Mammography screening rates were negatively associated with regional breast cancer incidence in non-stratified models (incident cases − 0.31 per % increase in mammography screening rate, 95% CI − 0.57, − 0.06, p = 0.02) and retained associations in urban county models. However, screening rates were only moderately associated with increased distant breast cancer incidence in non-stratified models (incident cases 0.12 cases per % increase in mammography screening rate, 95% CI 0.00, 0.24, p = 0.05) and were not associated with other breast cancer stages and did not retain associations in rural models (Table 1).

Interestingly, percent smokers within a county were at least moderately associated with decreased rates of localized breast cancer (incident cases − 1.14 per % increase in smokers, 95% CI − 2.41, − 0.12, p = 0.08) in non-stratified and urban county models, and increased rates in distant breast cancer in urban county models only (incident cases 0.29 per % increase in smokers, 95% CI − 0.01, 0.61, p = 0.06) (Table 1).

### Poor environmental quality is associated with increased breast cancer incidence

**Figure 2:**
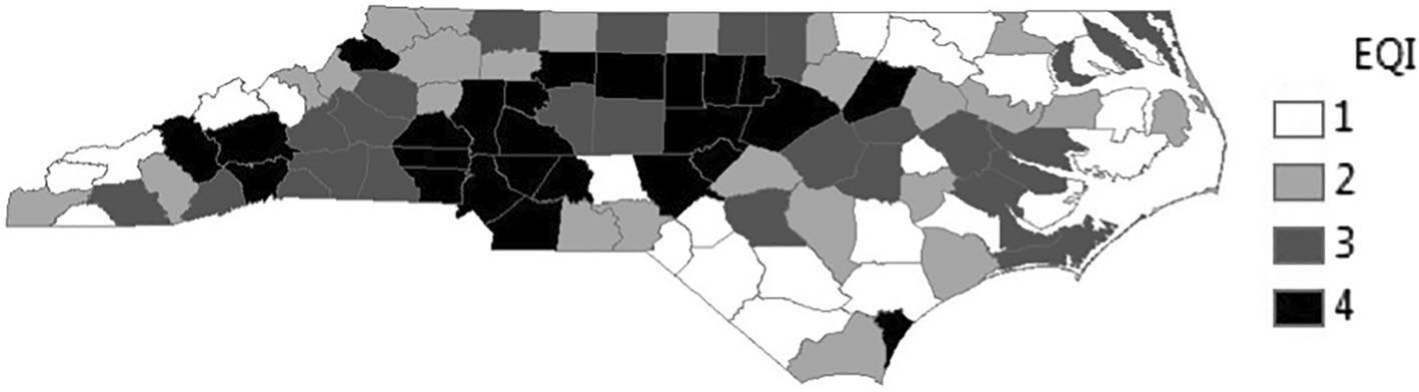
Environmental quality distribution in North Carolina. Overall Environmental Quality Index (EQI) data for North Carolina from the 2000–2005 dataset, quartiled and reported for each county. Overall EQI incorporates all data inputs for air, water, land, sociodemographic, and built environment domains. Data sourced from the U.S. Environmental Protection Agency.

Environmental quality is variable across the State of North Carolina (Fig. 2), akin to variability across the United States, as the interquartile range (25th–75th percentile) of total EQI in NC is − 0.187 to 0.734 while the interquartile range is − 0.606 to 0.706 for the US, making NC EQI analyses generalizable to a number of states and counties across the U.S., although what drives poor environmental quality varied by region and by county. The worst environmental quality (4th quartile) is present primarily in the central and western portions of North Carolina, like patterning seen in total breast cancer significantly high standardized incidence ratios (Fig. 1A).

In areas with poor overall environmental quality, total (10.82 incident cases, 95% CI 2.04, 19.60, p = 0.02) and localized breast cancer incidence (5.59 incident cases, 95% CI 0.59, 10.58, p = 0.03) was higher than in areas with good environmental quality (Table 2). Only distant breast cancer decreased with poorer environmental quality. This signifies that in counties with poor environmental quality, there were an average of 10.82 additional total breast cancer cases per 100,000 people, and specifically 5.59 extra localized breast cancer cases, compared to counties with good environmental quality.

**Table 2:** Generalized linear model estimates and associated p-values for poor EQI (third and fourth quartiles) as compared to good EQI (first and second quartiles).

Environmental Quality (REF = GOOD)		Non-stratified		Urban		Rural	
		*Estimate (95% CI)*	*p-value*	*Estimate (95% CI)*	*p-value*	*Estimate (95% CI)*	*p-value*
Total							
Total EQI	Poor	**10.82 (2.04, 19.60)**	**0.02**	**11.47 (1.46, 21.48)**	**0.03**	9.04 (− 7.96, 26.04)	0.29
Air domain	Poor	4.98 (− 4.19, 14.16)	0.28	− 0.30 (− 10.73, 10.12)	0.95	12.61 (− 8.47, 33.69)	0.23
Water domain	Poor	− 3.25 (− 11.19, 4.68)	0.42	− 0.25 (− 9.23, 8.72)	0.95	− 6.79 (− 21.44, 7.84)	0.35
Land domain	Poor	**11.44 (3.01, 19.87)**	**0.01**	**10.90 (0.85, 20.95)**	**0.03**	8.62 (− 7.44, 24.69)	0.28
Sociodemographic domain	Poor	0.30 (− 10.53, 11.14)	0.96	4.24 (− 10.80, 19.29)	0.57	− 14.50 (− 36.02, 7.02)	0.18
Built domain	Poor	6.53 (− 1.90, 14.96)	0.13	6.53 (− 3.70, 16.77)	0.21	3.29 (− 12.36, 18.95)	0.67
In situ							
Total EQI	Poor	1.22 (− 1.88, 4.33)	0.44	1.97 (− 2.31, 6.25)	0.36	0.57 (− 4.78, 5.93)	0.83
Air domain	Poor	− 1.50 (− 4.63, 1.61)	0.34	− 3.37 (− 7.43, 0.67)	0.10	3.76 (− 2.77, 10.29)	0.25
Water domain	Poor	− 2.71 (− 5.43, 0.00)	0.05	− 2.84 (− 6.33, 0.64)	0.11	− 1.69 (− 6.52, 3.12)	0.48
Land domain	Poor	**5.25 (2.34, 8.16)**	**0.00**	**5.46 (1.55, 9.37)**	**0.01**	4.72 (− 0.36, 9.82)	0.07
Sociodemographic domain	Poor	1.70 (− 1.92, 5.34)	0.35	2.81 (− 3.03, 8.67)	0.34	− 3.39 (− 10.07, 3.29)	0.31
Built domain	Poor	2.79 (− 0.10, 5.70)	0.06	**4.50 (0.52, 8.48)**	**0.03**	− 0.24 (− 5.25, 4.76)	0.92
Localized							
Total EQI	Poor	**5.59 (0.59, 10.58)**	**0.03**	**6.79 (1.14, 12.45)**	**0.02**	3.06 (− 7.22, 13.35)	0.55
Air domain	Poor	3.08 (− 2.08, 8.25)	0.24	2.13 (− 3.76, 8.04)	0.47	4.90 (− 7.41, 17.21)	0.42
Water domain	Poor	− 2.26 (− 6.73, 2.20)	0.32	1.26 (− 3.81, 6.35)	0.62	− 6.27 (− 14.82, 2.27)	0.15
Land domain	Poor	**6.98 (2.24, 11.73)**	**0.00**	**6.02 (0.33, 11.71)**	**0.04**	6.22 (− 3.15, 15.61)	0.19
Sociodemographic domain	Poor	− 1.65 (− 7.75, 4.44)	0.59	− 0.43 (− 8.95, 8.09)	0.92	− 6.42 (− 18.99, 6.14)	0.31
Built domain	Poor	3.55 (− 1.19, 8.30)	0.14	1.13 (− 4.66, 6.93)	0.70	4.77 (− 4.36, 13.92)	0.30
Regional							
Total EQI	Poor	− 0.13 (− 3.29, 3.03)	0.93	2.18 (− 0.78, 5.16)	0.15	− 0.83 (− 7.68, 6.02)	0.81
Air domain	Poor	0.48 (− 2.89, 3.87)	0.78	1.66 (− 1.50, 4.82)	0.30	− 2.21 (− 10.96, 6.52)	0.61
Water domain	Poor	− 0.78 (− 3.70, 2.14)	0.60	0.26 (− 2.45, 2.99)	0.84	− 2.75 (− 8.82, 3.31)	0.36
Land domain	Poor	0.28 (− 2.82, 3.38)	0.86	− 0.88 (− 3.93, 2.15)	0.56	0.96 (− 5.69, 7.63)	0.77
Sociodemographic domain	Poor	− 3.01 (− 7.00, 0.98)	0.14	− 0.93 (− 5.50, 3.62)	0.68	− 3.76 (− 12.69, 5.15)	0.40
Built domain	Poor	1.32 (− 1.78, 4.43)	0.40	0.11 (− 2.98, 3.22)	0.94	0.96 (− 5.53, 7.45)	0.77
Distant							
Total EQI	Poor	− **1.24 (**− **2.45, **− **0.02)**	**0.05**	− 0.85 (− 2.23, 0.52)	0.22	− 1.10 (− 3.83, 1.61)	0.41
Air domain	Poor	− **1.31 (**− **2.51, **− **0.10)**	**0.03**	− 1.21 (− 2.61, 0.18)	0.09	− 0.22 (− 3.30, 2.86)	0.88
Water domain	Poor	0.09 (− 0.96, 1.15)	0.86	0.58 (− 0.57, 1.73)	0.31	0.07 (− 2.68, 2.82)	0.96
Land domain	Poor	0.49 (− 0.67, 1.66)	0.40	0.50 (− 0.80, 1.81)	0.44	− 0.59 (− 3.60, 2.41)	0.68
Sociodemographic domain	Poor	− 0.59 (− 2.11, 0.92)	0.43	0.82 (− 1.36, 3.02)	0.45	− 2.42 (− 6.11, 1.26)	0.18
Built domain	Poor	− 0.16 (− 1.34, 1.02)	0.79	0.10 (− 1.23, 1.43)	0.88	0.00 (− 3.18, 3.18)	1.00

Results are stratified by breast cancer stage and urbanicity. Bolded text indicates statistically significant estimates (p < 0.05).

**Figure 3:**
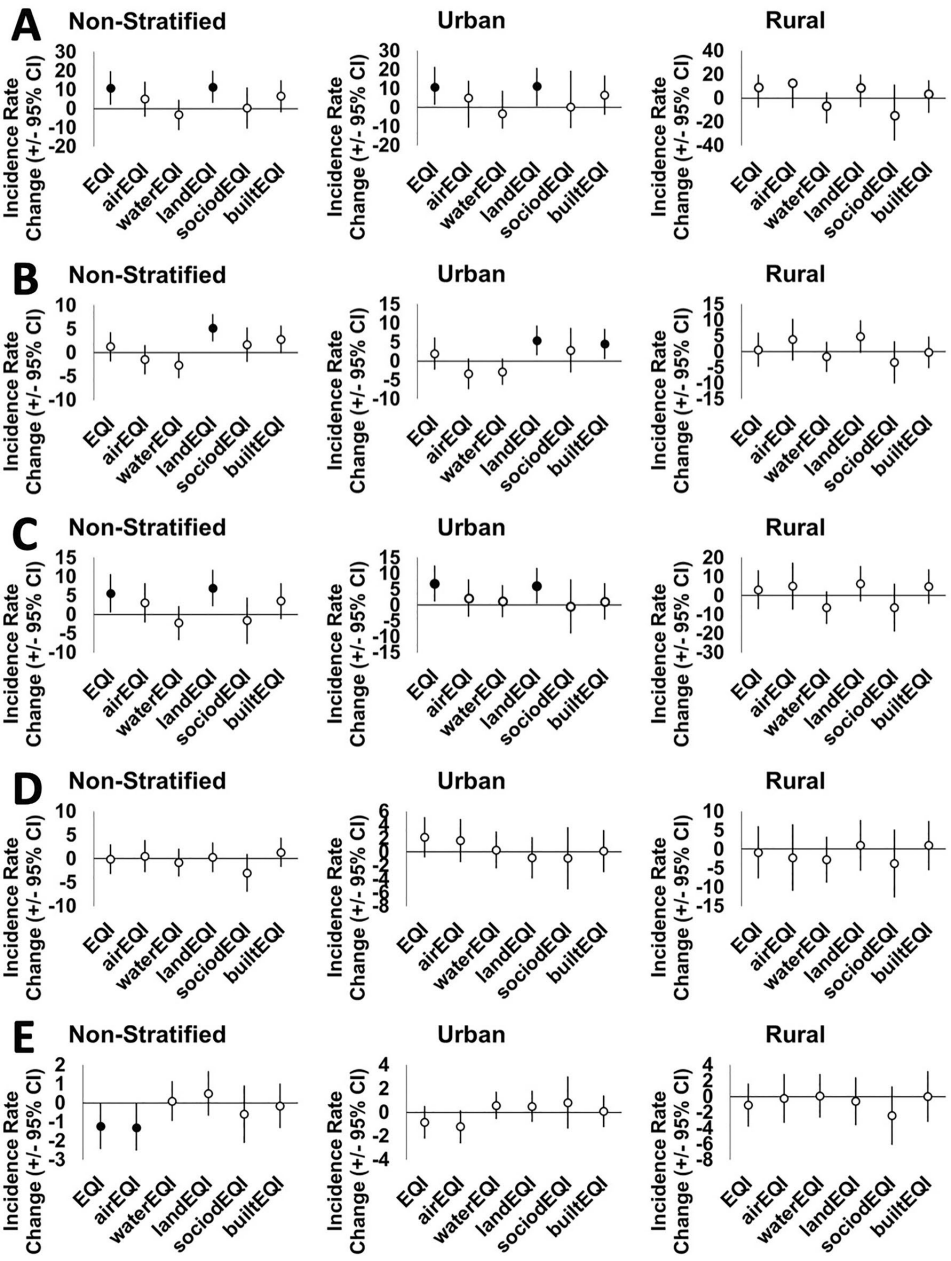
Environmental quality and rural–urban effects on breast cancer incidence rates of different stages. Estimates with 95% CI based on quartiled total and domain specific environmental quality index values for (**A**) total, (**B**) in situ, (**C**) localized, (**D**) regional, or (**E**) distant breast cancer incidence rates. Non-stratified and urban/rural category strata, good environmental quality (first and second quartiles) is reference and models adjusted for county-level percent smokers, percent AA, and mammography screening rates. Filled circles represent significant rate differences (p < 0.05).

Since breast cancer incidence was variable across North Carolina (Fig. 1, Supplemental Table S2), suggesting that the effect of environmental quality on breast cancer may differ in urban and rural communities, we stratified models by urbanicity. The association between poor environmental quality and increased breast cancer incidence remained in urban county models but not rural county models. For example, total breast cancer incidence was significantly higher in urban counties with poor environmental quality (11.47 incident cases, 95% CI 1.46, 21.48, p = 0.03, but was not significantly higher in rural counties with poor environmental quality (9.04 incident cases, 95% CI − 7.96, 26.04, p = 0.29) (Table 2, Fig. 3).

### Domain-specific environmental quality associations with breast cancer incidence varies by domain, breast cancer summary stage, and rural–urban context

Domain-specific environmental quality indices were associated with county-level breast cancer incidence rates differentially by summary stage (Table 2, Fig. 3). Most significantly, poor land environmental quality was positively associated with total (11.44 incident cases, 95% CI 3.01, 19.87, p = 0.00), carcinoma in situ (incident cases 5.25, 95% CI 2.34, 8.16, p = 0.00), and localized breast cancer incidence (incident cases 6.98, 95% CI 2.24, 11.73, p = 0.00). Poor air quality was associated with decreased distant breast cancer (incident cases − 1.31, 95% CI − 2.51, − 0.10, p = 0.03), while built environmental quality only had associations with carcinoma in situ that approached significance (incident cases 2.79, 95% CI − 0.10, 5.70, p = 0.06). Neither sociodemographic nor water environmental quality had significant associations with breast cancer incidence for any summary stage.

Associations between environmental quality and summary stages also differed by urbanicity. Associations between poor land environmental quality and increased total, carcinoma in situ, and localized breast cancer incidence remained significant in urban county models but only approached significance in rural county models for carcinoma in situ, wherein urban estimates were much higher than rural estimates. Poor air quality association with decreased distant breast cancer approached significance in urban county but not rural county models, while associations between built environmental quality and carcinoma in situ were increased and significant in urban county models (p = 0.03) but not rural county models (Table 2).

## Discussion

North Carolina has a population of over 10 million people spread across 100 different counties and is highly diverse in terms of race/ethnicity makeup, urbanicity, socioeconomic status, and in distribution of disease such as invasive breast cancer^39,40^. Our data also showed clear heterogeneity in both breast cancer incidence and environmental quality across the state, and SIR patterning varied geographically by breast cancer summary stage, suggesting that factors such as environmental quality which contribute to breast cancer vary geospatially.

The EQI provides a unique opportunity to explore cancer and environment associations in combination with stage-specific breast cancer patient data, as it has previously been utilized for high-level association studies with various cancer types. Our previous study using the EQI explored its associations with disease invasiveness among individuals with breast cancer, taking into account individual level factors (patient age, race, smoking status). A crucial aspect of the study design was to consider the heterogeneity of potential environmental impacts by breast tumor stage, environmental domain, and urbanicity. In complement, this study elucidated the community level effects of EQI exposures, in particular how they are associated with the development and incidence of different stages of breast cancer, while taking into account urbanicity and community factors (county percent African American, smokers, mammography screening rates).

Although we did not perform adjustment for multiple comparisons^48^ for statistical significance, we compare patterns and precision of estimates in order to better analyze trends. Our results indicate an association between poor environmental quality and higher total breast cancer incidence. We further observed positive associations between the land EQI and carcinoma in situ, localized, and total breast cancer incidence, primarily in the urban setting. Such positive associations have been shown previously with specific environmental exposures such as tobacco smoke, pesticides, and other environmental contaminants^12,14,16,17,19–21,49,50^. In addition, we see that the association between breast cancer and the environment varies by summary stage of breast cancer and environmental domain, suggesting that research grouping all breast cancer stages together into a total breast cancer number does not capture the full picture of how environmental exposures impact early versus late stages of breast cancer.

According to previous literature, there is a correlation between incidence of late-stage and aggressive subtypes of breast cancer and demographic factors such as socioeconomic status and urbanicity^45,51–57^. Indeed, previous investigations using the EQI also showed strong positive associations between cancer incidence and the EQI that differed by rural–urban status^36^, suggesting that urbanicity is a significant factor in cancer and environment interactions. Our sensitivity analysis investigating rural–urban difference shows that associations between breast cancer and the environment can differ between rural and urban contexts. This supports previous findings that rural–urban disparities exist in breast cancer^44,45,51^, in particular that environmental exposures can often have larger effects in urban areas^36^. It also suggests that individual factors driving poor environmental quality and associated with higher breast cancer incidence in urban areas may be different than those factors involved in these associations in rural areas.

Breast cancer incidence rates were higher for later stage disease (regional or distant) and total breast cancer in association with larger county percentages of African Americans. These results are consistent with past studies indicating African American race is a risk factor for later stage breast cancers, including being diagnosed at a later stage^3,52,58^. Similarly, in our analyses, higher mammography screening rates were associated with lower regional breast cancer incidence rates which was expected since higher screening rates are thought to decrease later stage diagnoses^41^. The opposite trend was seen in non-stratified distant breast cancer models, with moderately higher incidence associated with mammography screening rates, which was surprising. It was further surprising that counties with a higher percentage of smokers had a moderately lower incidence of localized breast cancers, since smoking has been associated with breast cancer risk previously and was associated with a moderately higher rate of distant breast cancer incidence in urban counties^12,59,60^. It is important to note that we did not have individual level smoking data, thus we do not know whether the women diagnosed with breast cancer in our study were smokers. Thus, we caution against the over interpretation of this result.

Our results showing significant associations between environmental quality and early-stage breast cancer raise an interesting question, namely why we do not see these same associations with more aggressive stages of disease. One potential drawback to our data is that regional is a stage which incorporates many clinically distinct substages, which includes lymph nodes, no lymph nodes, and/or direct extensions. Unfortunately, TNM staging information was unavailable for the majority of patients in the dataset, although future data years may incorporate these and allow for distinction by TNM stage rather than summary stage. In addition, a number of counties had low case numbers for the distant breast cancer summary stage and thus unstable and unreportable incidence rate estimates to use in our models, limiting our analyses.

Another limitation in our analysis includes using county at diagnosis, which may not be the most relevant for environmental exposures if patients did not live in that county when they were exposed to potentially important environmental factors. While residential mobility may occur, this would likely result in an exposure misclassification, likely biasing associations with breast cancer toward the null (i.e., no association). Future studies may be strengthened by detailed residential history data, since there are known periods of sensitivity and susceptibility in which environmental exposures impact breast cancer risk^61,62^. In addition, future studies would also benefit from individual-level patient data and more detailed environmental exposures data to circumvent the limitation of using county-level ecological and demographics data and allow for more refined statistical analysis.

The utility of the EQI includes the quantity of data, as well as its stratification into different environmental domains and by county urbanicity. This has allowed for a broader look herein at breast cancer and environment associations. It is again reiterated that delving deeper into specific associations was not possible in the present study given the aggregate-level data, yet it provides valuable information for future studies. In addition, a major strength of our analysis lies in using EQI and breast cancer patient data from North Carolina as a study site, given its range of population densities and environmental conditions, as well as its comprehensive central cancer registry, which manages and provided all cancer incidence data for this analysis.

The results found are significant, suggesting an association between land quality and early-stage breast cancer especially in urban counties, wherein land quality may be driven by factors such as pesticide usage, toxic releases including heavy metals, and polluted facilities including animal facilities^24^. Some studies have already linked factors such as pesticides to breast cancer^13–16,63^, and future studies should continue to explore environmental factors from the land domain and their potential associations with breast cancer, particularly early stages. These results are applicable across many parts of the U.S. with EQI ranges comparable to North Carolina, and similar methodologies could be utilized to investigate breast cancer and environment associations in other states.

## Conclusions

Our analysis suggests an association between breast cancer and the environment, particularly the land environment and early breast cancer stages, most pronounced in urban locations. This study further emphasizes that the impacts of environmental exposures can differ by breast cancer stage and urbanicity. These findings elucidate that investigating environmental impacts on total breast cancer may hide impacts on different stages of the disease, which presents a challenge in identifying actionable environmental risk factors for breast cancer, a highly heterogeneous disease. Including the EQI in future models investigating cancer and environment associations should be considered to control for confounding effects by the myriad exposures documented in the cumulative environment.

### Supplementary Information

Supplementary Information.

## Data Availability

Data for this study can be made available upon request by corresponding author Gayathri R. Devi, Ph.D., at gayathri.devi@duke.edu.
