# Supplementary material for: Cumulative environmental quality is associated with breast cancer incidence differentially by summary stage and urbanicity
Source: Sci Rep. 2023 Nov 20;13:20301. doi: 10.1038/s41598-023-45693-0 (PMC10662118; doi:10.1038/s41598-023-45693-0)
Supplement: Supplementary file 1 — Supplementary Information. [file 41598_2023_45693_MOESM1_ESM.docx]

**Cumulative environmental quality is associated with breast cancer incidence differentially by summary stage and urbanicity**

Supplemental Information

**Supplemental Table 1. Derived Summary Staging as Defined by NC CCR, adapted from SEER**

| **Breast Cancer Stage** | **SEER Derived Summary Staging** | **Definition** |
| --- | --- | --- |
| In situ | In situ | Noninvasive; intraepithelial; Intraductal WITHOUT infiltration; Lobular neoplasia; Noninfiltrating; In situ Paget disease |
| Localized | Localized | Confined to breast tissue and fat including nipple and/or areola, Paget disease WITH or WITHOUT underlying tumor; Localized, NOS |
| Regional | Regional by direct extension only | Attachment or fixation to pectoral muscle or underlying tissue Deep fixation Extensive skin involvement: En cuirasse Erythema Inflammation of skin Lenticular nodule(s) Peau d’orange (skin of orange) “Pigskin” Satellite nodule(s) in skin of primary breast Skin edema Ulceration of skin of breast Inflammatory carcinoma, including diffuse (beyond that directly overlying the tumor) dermal lymphatic permeation or infiltration### Invasion of (or fixation to): Chest wall Intercostal muscle(s) Pectoral fascia or muscle(s) Rib(s) Serratus anterior muscle(s) Subcutaneous tissue Local infiltration of dermal lymphatics adjacent to primary tumor involving skin by direct extension Skin infiltration of primary breast including skin of nipple and/or areola |
|  | Ipsilateral regional lymph node(s) involved only | REGIONAL Lymph Nodes Axillary, NOS: Level I (low) (superficial), NOS [adjacent to tail of breast]: Anterior (pectoral) Lateral (brachial) Posterior (subscapular) Level II (mid-level) (central), NOS: Interpectoral; (Rotter’s) Level III (high) (deep), NOS: Apical (subclavian) Axillary vein Infraclavicular ###*** (subclavicular) Internal mammary (parasternal) Intramammary Nodule(s) in axillary fat Regional lymph node(s), NOS |
|  | Regional by BOTH direct extension AND ipsilateral regional lymph node(s) involved | Codes Regional by direct extension only + Ipsilateral regional lymph node(s) involved only |
|  | Regional, NOS | Regional, NOS |
| Distant | Distant | Distant lymph node(s): Cervical, NOS Contralateral/bilateral axillary Contralateral/bilateral internal mammary (parasternal) Supraclavicular (transverse cervical) Other distant lymph node(s) Further contiguous extension: Skin over: Axilla Contralateral (opposite) breast Sternum Upper abdomen; Metastasis: Adrenal (suprarenal) gland Bone other than adjacent rib Contralateral (opposite) breast - if stated as metastatic Lung Ovary Satellite nodule(s) in skin other than primary breast |

**Supplemental Table 2. Average, median, and interquartile range of breast cancer incidence rates by summary stage for rural and urban counties in NC**

|  | **Rural** | | | | | **Urban** | | | | |
| --- | --- | --- | --- | --- | --- | --- | --- | --- | --- | --- |
|  | **Total** | **In situ** | **Localized** | **Regional** | **Distant** | **Total** | **In situ** | **Localized** | **Regional** | **Distant** |
| **Average** | 148.7 | 25.0 | 80.5 | 41.5 | 8.4 | 157.2 | 25.5 | 84.3 | 40.7 | 7.1 |
| **Median** | 151.2 | 23.8 | 80.5 | 40.7 | 8.6 | 160.6 | 24.6 | 84.7 | 40.4 | 6.9 |
| **25%** | 137.7 | 20.0 | 73.7 | 36.5 | 6.5 | 148.5 | 20.3 | 77.8 | 38.4 | 5.7 |
| **75%** | 167.8 | 29.4 | 89.4 | 45.5 | 9.8 | 168.8 | 30.7 | 92.5 | 43.4 | 8.1 |
| **IQR** | 30.2 | 9.5 | 15.8 | 9.0 | 3.3 | 20.3 | 10.4 | 14.7 | 5.0 | 2.4 |
| ***p-value Rural:Urban*** | *0.049* | *0.718* | *0.135* | *0.611* | *0.074* |  |  |  |  |  |

**Supplemental Figure 1. Age-Standardized Breast Cancer Incidence Rates by County**

Quartiled annual age-standardized incidence rates for (A) total, (B) in situ carcinoma, (C) localized, (D) regional, and (E) distant breast cancer in North Carolina, 2010-2014. Data sourced from the NC Central Cancer Registry, adjusted to the US 2000 Census.
